# QuickStats

**Published:** 2014-08-01

**Authors:** FROM THE NATIONAL CENTER FOR HEALTH STATISTICS

**Figure:**
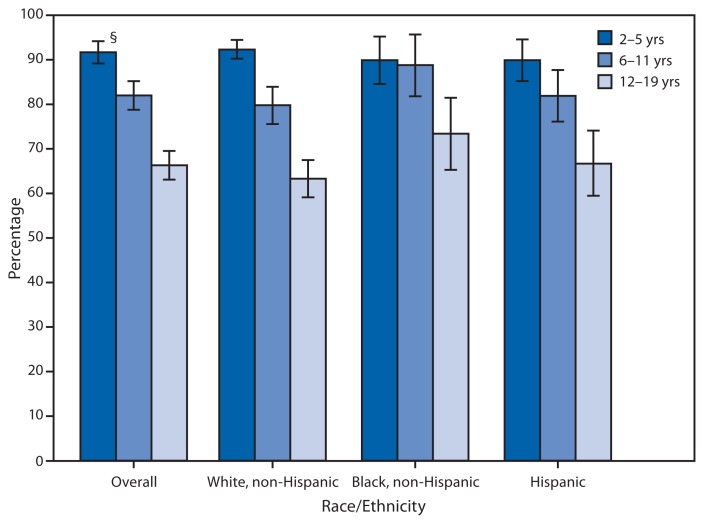
Percentage of Youths Who Consumed Fruit* on a Given Day,^†^ by Age Group and Race/Ethnicity — National Health and Nutrition Examination Survey, United States, 2009–2010 * The definition of fruit comes from the U.S. Department of Agriculture’s Food Patterns Equivalents Database and includes both fruit and fruit juices. ^†^ The National Health and Nutrition Examination Survey collects dietary intake information using 24-hour dietary recall interviews. ^§^ 95% confidence interval.

During 2009–2010, among youths overall aged 2–19 years, 91.7% of those aged 2–5 years, 82.0% of those aged 6–11 years, and 66.3% of those aged 12–19 years were reported as consuming fruit on a given day. Among non-Hispanic white, non-Hispanic black, and Hispanic youths, the percentage who consumed fruit among those aged 2–5 years was significantly greater than the percentage among those aged 12–19 years. Among those aged 2–5 years, the percentage who consumed fruit was 92.3% for non-Hispanic white youths, 89.9% for non-Hispanic black youths, and 89.9% for Hispanic youths; in contrast, among those aged 12–19 years, the percentage was 63.3% for non-Hispanic whites, 73.4% for non-Hispanic blacks, and 66.7% for Hispanics.

**Source:** Nielsen SJ, Rossen LM, Harris DM, Ogden CL. Fruit and vegetable consumption of US youth, 2009–2010. NCHS data brief no. 156. Hyattsville, MD: US Department of Health and Human Services, CDC; 2014. Available at http://www.cdc.gov/nchs/data/databriefs/db156.htm.

**Reported by:** Samara Joy Nielsen, PhD, snielsen@cdc.gov, 301-458-4193; Steven M. Frenk, PhD.

